# The mitogenome portrait of Umbria in Central Italy as depicted by contemporary inhabitants and pre-Roman remains

**DOI:** 10.1038/s41598-020-67445-0

**Published:** 2020-07-01

**Authors:** Alessandra Modi, Hovirag Lancioni, Irene Cardinali, Marco R. Capodiferro, Nicola Rambaldi Migliore, Abir Hussein, Christina Strobl, Martin Bodner, Lisa Schnaller, Catarina Xavier, Ermanno Rizzi, Laura Bonomi Ponzi, Stefania Vai, Alessandro Raveane, Bruno Cavadas, Ornella Semino, Antonio Torroni, Anna Olivieri, Martina Lari, Luisa Pereira, Walther Parson, David Caramelli, Alessandro Achilli

**Affiliations:** 1 Department of Biology, University of Florence, 50122 Florence, Italy; 2 Department of Chemistry, Biology and Biotechnology, University of Perugia, 06123 Perugia, Italy; 3 Department of Biology and Biotechnology “L. Spallanzani”, University of Pavia, 27100 Pavia, Italy; 4 Institute of Legal Medicine, Medical University of Innsbruck, 6020 Innsbruck, Austria; 5 Istituto di Tecnologie Biomediche, CNR, Segrate, 20090 Milan, Italy; 6 M.A.N.U. National Archeological Museum of Umbria, 06121 Perugia, Italy; 7 IPATIMUP (Instituto de Patologia e Imunologia Molecular da Universidade do Porto), Porto, Portugal; 8 i3S (Instituto de Investigação e Inovação em Saúde, Universidade do Porto), 4200-135 Porto, Portugal; 9 Forensic Science Program, The Pennsylvania State University, University Park, PA 16801 USA

**Keywords:** Phylogenetics, Biological anthropology, Haplotypes, Population genetics

## Abstract

Umbria is located in Central Italy and took the name from its ancient inhabitants, the *Umbri*, whose origins are still debated. Here, we investigated the mitochondrial DNA (mtDNA) variation of 545 present-day Umbrians (with 198 entire mitogenomes) and 28 pre-Roman individuals (obtaining 19 ancient mtDNAs) excavated from the necropolis of *Plestia*. We found a rather homogeneous distribution of western Eurasian lineages across the region, with few notable exceptions. Contemporary inhabitants of the eastern part, delimited by the Tiber River and the Apennine Mountains, manifest a peculiar mitochondrial proximity to central-eastern Europeans, mainly due to haplogroups U4 and U5a, and an overrepresentation of J (30%) similar to the pre-Roman remains, also excavated in East Umbria. Local genetic continuities are further attested to by six terminal branches (H1e1, J1c3, J2b1, U2e2a, U8b1b1 and K1a4a) shared between ancient and modern mitogenomes. Eventually, we identified multiple inputs from various population sources that likely shaped the mitochondrial gene pool of ancient *Umbri* over time, since early Neolithic, including gene flows with central-eastern Europe. This diachronic mtDNA portrait of Umbria fits well with the genome-wide population structure identified on the entire peninsula and with historical sources that list the *Umbri* among the most ancient Italic populations.

## Introduction

Due to its acknowledged potential, archaeogenetics is broadly applied to study ancient civilizations, demographic histories and migration events. Markedly, advances in high-throughput genotyping technology have highlighted how the present-day genetic variation of human populations is the outcome of past population movements. In prehistoric times, the Mediterranean area experienced three significant migration waves whose legacy is retrieved in the mitochondrial pool of modern and ancient populations: the Paleolithic hunter-gatherers who survived and re-expanded from glacial refuges, the Neolithic farming societies that moved from the East, and the herders from the Pontic-Caspian steppes inaugurating the Bronze Age^1–13^.

In this scenario, the Italian Peninsula played a pivotal role in human migrations around the Mediterranean Sea, as testified by the higher degree of its current genomic variability compared with other European populations^14–19^. This complexity is the result of multifaceted inputs that shaped its gene pool since the Upper Paleolithic. Inferring the contributions of each process is further complicated by similar (or partially overlapping) dispersal patterns from, to and even within the Italian Peninsula, often separated by short time frames. It is generally agreed that the ancestral contribution came from the ancient Italic peoples, among which Latins (also called pre-Romans) achieved a dominant position establishing Roman civilization; whereas the invasions after the fall of the Roman Empire did not significantly alter the peninsular gene pool^18,19^.

Concerning the phylogeography of Italy, it is difficult to identify a clear genetic pattern able to discriminate southern, northern and central populations in spite of several attempts based on autosomal and uniparental markers^19–24^. Southern populations were mostly influenced by Greek and Arab colonizations, Northern Italians might reflect admixture with French and German-speaking populations, while Central Italy occupies its own intermediate position creating a continuous cline of variation across the peninsula (with Sardinians as outliers)^13,19,25–29^. Most of these studies were performed on a large geographic scale producing low-definition results and mainly focusing on modern populations. As for microgeographic studies on Central Italy, only Etruscans (in Tuscany) and Picentes (in Marche) were the target of specific analyses that highlighted their genetic affinity with the current inhabitants^30–37^. However, Umbria, another crucial region in Central Italy, is still unexplored. The name derives from the ancient Umbrians (or *Umbri*), traditionally considered an indigenous and very old population^38^. In the first century Common Era (CE) Pliny the Elder stated: “The population of Umbria is considered the oldest of Italy, and it is believed that the Umbrians had been called Ombrikòi by the Greeks because they survived the rains when the earth was flooded” (Pliny the Elder, *Naturalis Historia*, III, 112). Nevertheless, the origin and ethnic affinities of the Umbrians are still in some degree a matter of dispute.

Archaeological and historical data suggest that during the Early Iron Age (ninth/eighth centuries BCE, Before Common Era), Umbrians were among the first communities with strong and well-defined cultural identities in Central Italy, together with Etruscans (to the west), Picentes (to the east) and Samnites (to the south). They originally occupied the eastern part of the today’s Umbria region, placed on the left bank of the Tiber River, soon extending their territories in western Umbria and Tuscany. Around the sixth century BCE, the Etruscans, who had already begun to influence the Umbrian culture, took control over the western territories and the Tiber became the natural border between Umbrians and Etruscans^39^. The degree of interaction between these ancient populations is still unclear. The Romans came into contact for the first time with the Umbrians during the fourth century BCE and established Latin colonies in the area at the beginning of the third century BCE. After 260 BCE, Umbria was already under the full control of Rome^40^, while the Etruscan culture (and language) disappeared only at the time of the “Social War” (90-88 BCE) with the attribution of Roman citizenship to all Italic people^41^. Nowadays Umbria is somewhat smaller than ancient Umbria, but its inhabitants still preserve significant difference in the dialects spoken on the two banks of the Tiber^42^.

An important necropolis in East Umbria is placed in the so-called *Plestinam Paludem* (now Colfiorito, located at 760 m above the sea level up in the Apennines). The *Plestini* plateaus represented an obligatory way in the trans-Apennine routes, but stable settlements have not been attested before the beginning of the Iron Age^43,44^. The geographical position, the wealth of water, the possibilities offered by the exercise of hunting and fishing, the goodness of the pastures and the abundance of timber have undoubtedly encouraged the stabilization and growth of the population during the Iron Age.

**Figure 1:**
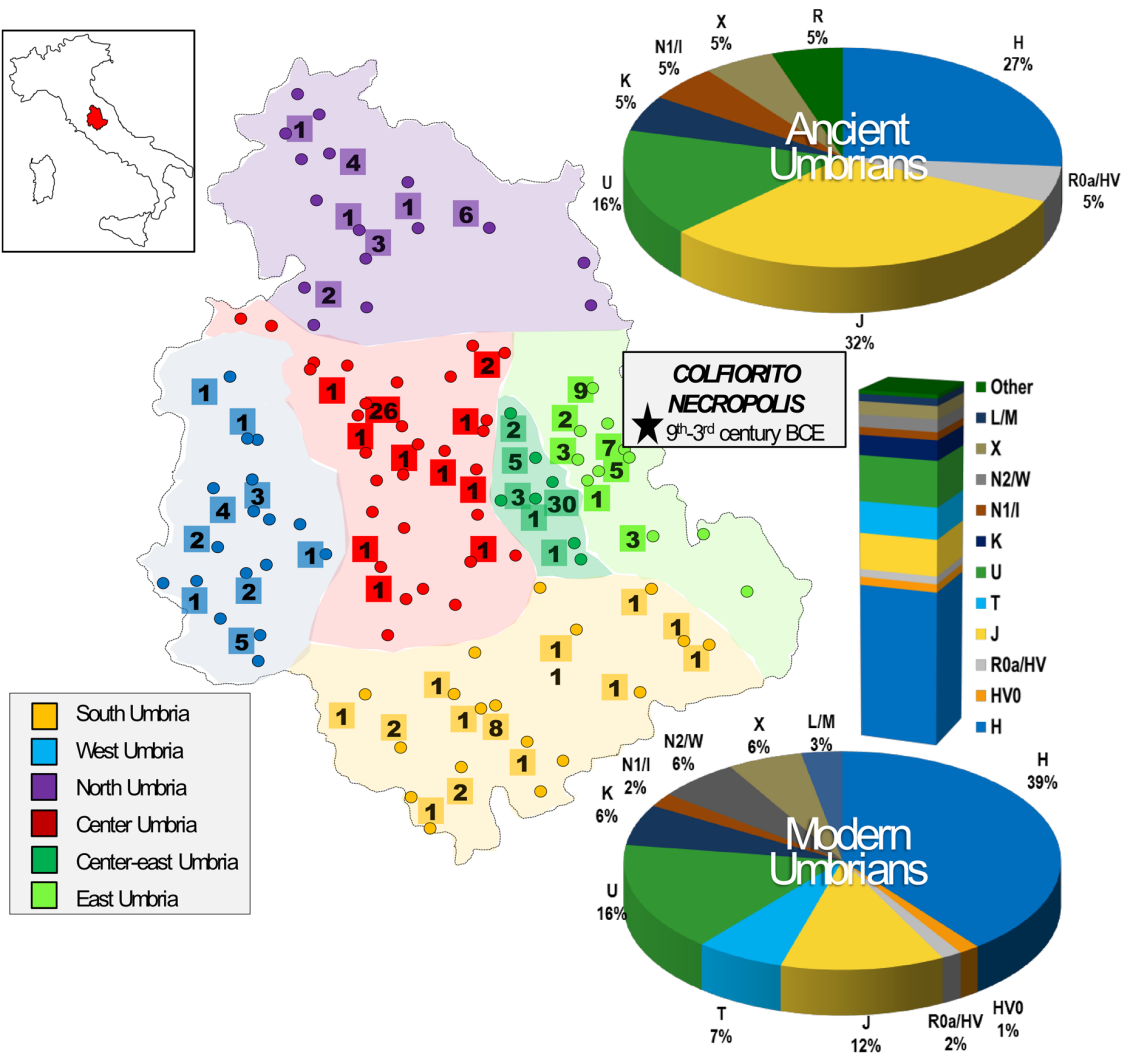
Geographic origin of ancient and modern Umbrians analyzed in this study. The six established sub-regions are symbolized by different colors. Dots mark the geographic origin of all modern samples (N = 545, see Supplementary Dataset S1); those completely sequenced are reported in squares (N = 198, see Supplementary Dataset S2). Pie charts summarize haplogroup distributions (based on Haplogrep) considering complete mitogenomes of ancient (N = 19, see Supplementary Dataset S3) and modern samples, while the bar plot represents control-region data of the overall modern sample. The location of the Colfiorito necropolis is indicated by a star.

In this study, we report 198 entire mitogenomes from modern Umbrians (191 here sequenced for the first time), selected from a larger dataset of 545 samples covering the entire region, as well as the mitogenomes of 19 Iron Age *Umbri Plestini*, who were buried in *Plestinam Paludem* (Fig. 1 and Supplementary Fig. S1). This diachronic approach allowed us to study the mitochondrial DNA (mtDNA) variation (at the highest-resolution level) in a microgeographic context and to obtain new insights concerning the maternal genetic history of Umbria, a region often defined as the “Heart of Italy” because of its location.

## Results and discussion

### Mitochondrial variation of modern Umbrians

#### Control-region data

**Figure 2:**
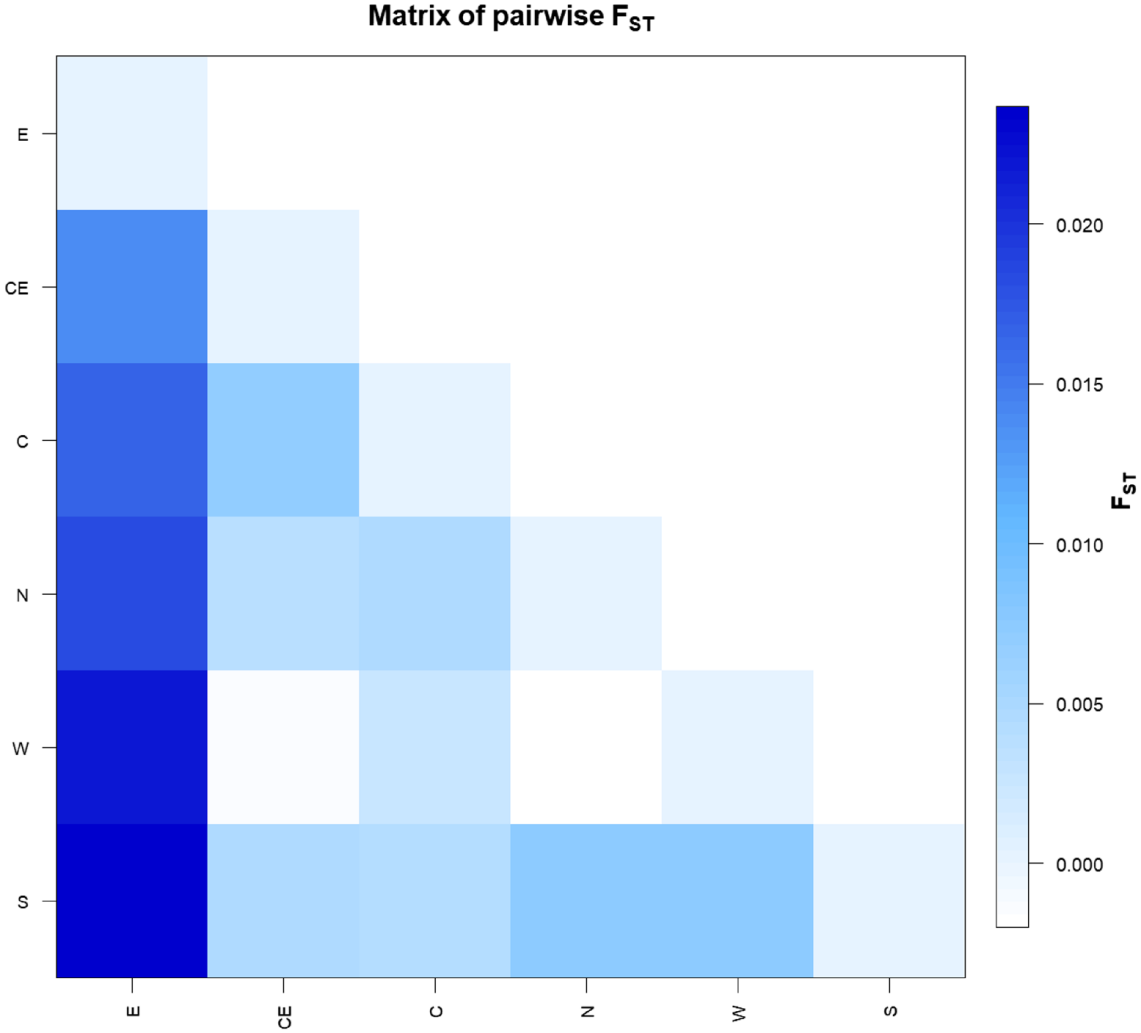
Pairwise population genetic distances. Plot of pairwise population genetic distances between the six established sub-regions of Umbria (E = east; CE = center-east; C = center; N = north; W = west; S = south), based on control-region data (n = 480).

Through the analysis of the control-region sequence of 545 modern Umbrians (Supplementary Dataset S1), it was possible to identify a high haplotype diversity (Hd = 0.994) that, compared to other Eurasian and North African populations^21^, confirms the goodness of the sampling and testifies for an extensive maternal admixture (Supplementary Fig. S2). In order to verify if this variability is equally distributed within the region without any sub-population differentiation, we estimated pairwise fixation index (*Fst*) values in six sub-areas, considering geographic and historical criteria (north, south, west, center, center-east and east; Fig. 1), showing that inhabitants from eastern Umbria are genetically the most distant from the other sub-groups (Fig. 2). This high differentiation of the eastern part of Umbria suggests a distinctiveness in its ancient or recent history compared to the rest of the region.

**Figure 3:**
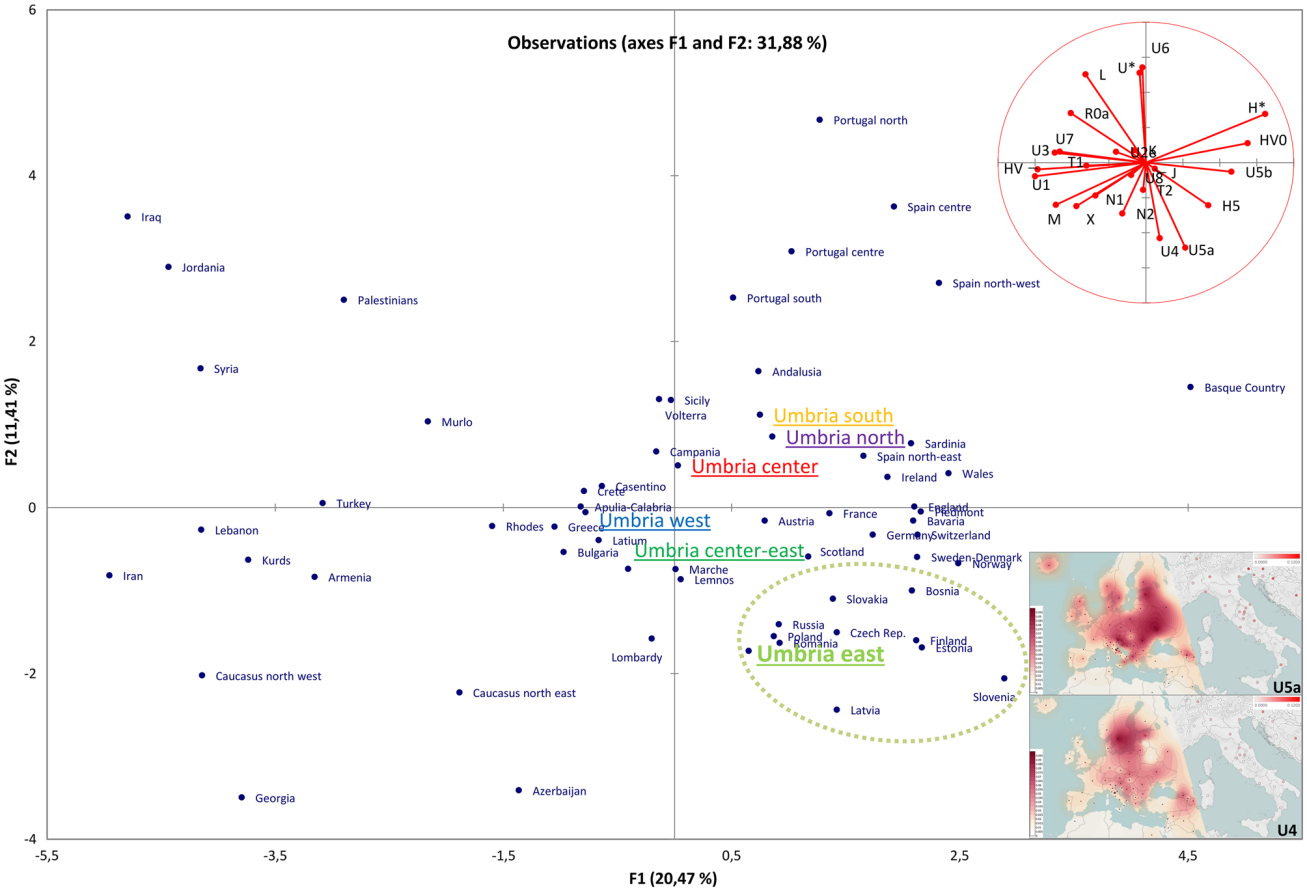
PCA plot. The genetic landscape of Eurasia based on haplogroup frequencies from control-region data. The inset shows the frequency distributions of U4 and U5a in western Eurasia (left side) as well as in Italy (right side) constructed with Tableau 2019.3.0 (www.tableau.com).

Phylogenetic analyses were then performed. The mutational motifs of the 545 Umbrians clustered into 369 haplotypes belonging to numerous haplogroups and sub-haplogroups when using Haplogrep 2.0 and SAM 2 on EMPOP (Supplementary Dataset S1). As expected, most (97%) are members of typical western Eurasian branches. Initially, we compared macro-haplogroup distributions among the six established sub-regions identifying two significant differences in haplogroups J, which is particularly common (30%) in East Umbria, and K, with a rather high incidence (17%) in South Umbria (Supplementary Fig. S3A). In order to summarize the information embedded in these haplogroups, we performed a principal component analysis (PCA, Fig. 3) including the six Umbrian sub-regions and the Eurasian dataset previously used to analyze the neighboring Tuscany region^30^. The relatedness of different parts of Umbria with typical Mediterranean populations can be clearly appreciated in the middle portion of the plot. However, East Umbria clusters together with eastern European countries. Major contributions to this clustering come from haplogroups U4 and U5a, which show high frequencies in central-eastern Europe (inset of Fig. 3). Notably, two of their sub-branches (U4a and U5a1) have been also identified in *Yamnaya* samples^2^ as well as in Mesolithic samples from northern and eastern Europe (Reich database V42.4; https://reich.hms.harvard.edu).

#### Complete mitogenome data

Taking the population density into account, we randomly selected samples (from 19 to 42) from each of the six regional divisions for complete mtDNA sequencing. With this approach we obtained 191 novel mitogenomes (Supplementary Dataset S2), selected considering only geographic criteria without any phylogenetic bias.

It is worth mentioning that we did not notice any difference when comparing the two NGS methodologies used to generate the complete mitogenomes. To check if any ascertainment bias was present, we performed a Site Frequency Spectrum (SFS) analysis, using the two methodologies as “artificial populations” and comparing the distributions of variant occurrences in the two datasets. As shown in Supplementary Figure S4, we observed a comparable amount of singletons and doubletons, which are used as indicators of possible inconsistencies.

Our mitogenomes, together with seven GenBank records (189 haplotypes in total), were classified into different sub-haplogroups (147 with Haplogrep and 137 with EMPOP). The frequencies of major haplogroups widely overlap with those obtained from the control-region dataset, without any significant differences (*p* value 0.57), thus confirming that even the 198 complete mitogenomes can be accounted as a population dataset representative of modern Umbrians. Moreover, also the macro-haplogroup distributions in the six sub-regions showed the same pattern of the control-region data, confirming significant differences only for haplogroups J and K in East and South Umbria, respectively (Supplementary Fig. S3B). On the other hand, the importance of complete mitogenome sequencing is confirmed by the increased haplotype diversity value (from 0.994 to 0.999) as well as by the accuracy of the sub-haplogroup classification, which was improved for more than 70% of haplotypes (76% for Haplogrep, 72% for EMPOP; Supplementary Dataset S2).

### MtDNA variation of ancient Umbrians

Using NGS technology combined with target enrichment^45^, we tried to reconstruct the mitogenomes of 28 pre-Roman samples from the necropolis of *Plestia*, located in East Umbria (Fig. 1 and Supplementary Fig. S1). Four direct radiocarbon dates confirmed the age estimated from the archaeological context placing the remains at the end of the seventh cal. century BCE (Supplementary Fig. S5). Eventually, four of the 28 samples did not amplify at all, while five produced ambiguous sequencing results that did not reach the standard quality requested to guarantee the reliability of NGS data (Supplementary Fig. S1). The final dataset of 19 ancient mitogenomes showed a depth of average coverage ranging from 5.86× to 50.98× (Supplementary Dataset S3). The damage pattern and average fragment size were used in an iterative probabilistic approach that jointly estimates modern human contaminations and reconstructs the endogenous mtDNA sequence^46^. Nucleotide misincorporations and fragmentation patterns were compatible with the sample age^47^, ranging between 16.7 and 42.1% at 5′ molecule termini and 60.57–100.41 bps, respectively. In addition, no significant levels of contamination were detected.

**Figure 4:**
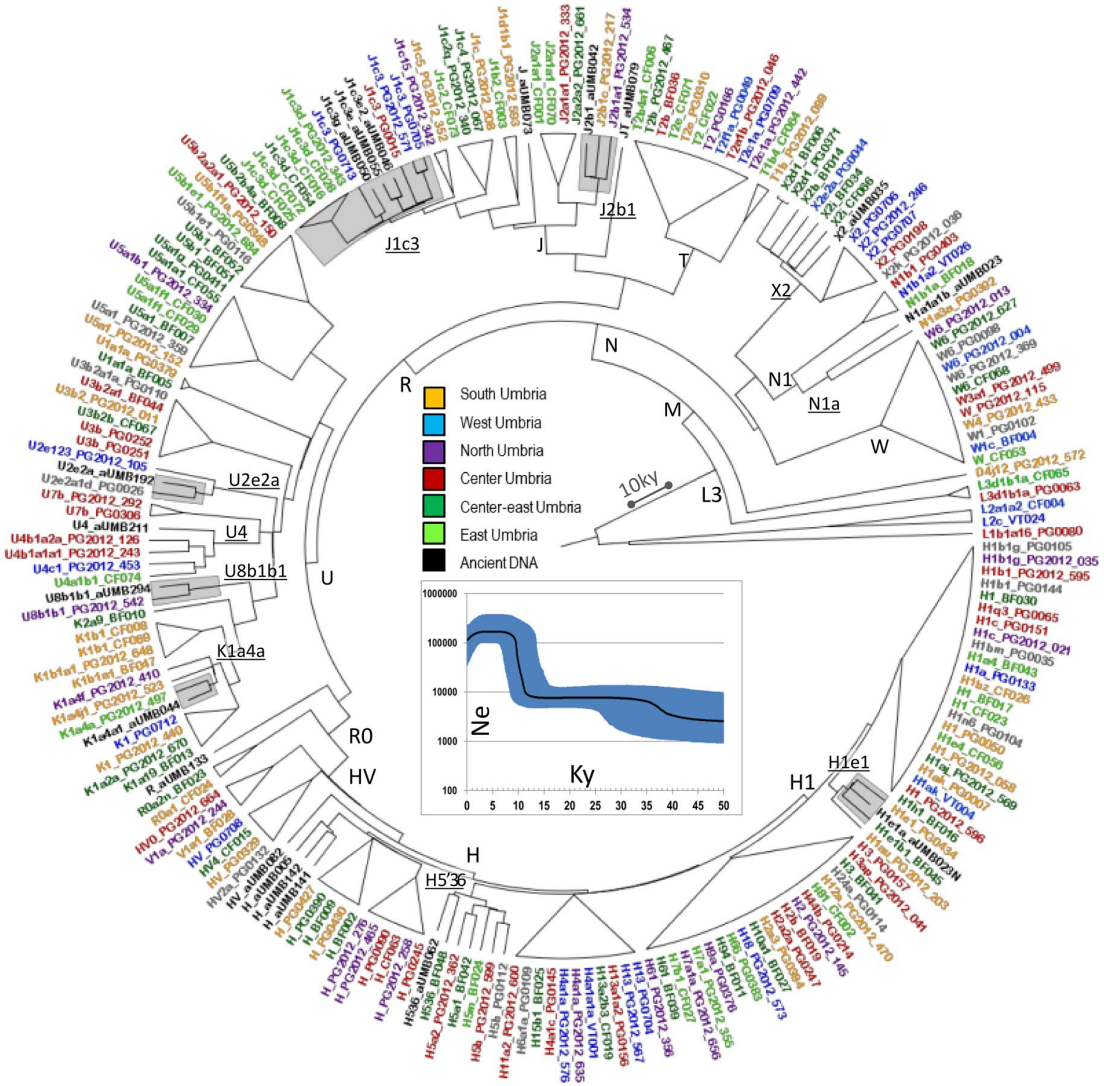
Schematic phylogenetic tree of modern and ancient Umbrian mitogenomes. The terminal branches shared between ancient and modern mtDNAs are shaded. Branch lengths are drawn to scale based on Bayesian time estimates. The inset shows a Bayesian Skyline Plot (BSP) analysis of Umbrian mitogenomes. See Supplementary Figure S6 for details.

The 19 mtDNA sequences were classified into 17 mitochondrial haplogroups and eight super-haplogroups. They are all typical of present-day West-Eurasian populations with the most represented lineage being J (32%), followed by H (26%) and U (16%) (Figs. 1, 4). A similar H frequency (~ 30%) was observed in modern samples from the eastern part of the region. Haplogroup H is the most frequent in Europe (~ 40%) with a declining pattern from western Europe towards the Near East and Caucasus (~ 10–20%), but without any conclusive scenario about its still enigmatic origin^48^. Regarding the most represented haplogroup J (three mitogenomes belonging to different subsets of J1c3), it has been proposed that most of its subgroups diversified in the Near East during the Last Glacial Maximum (LGM) and spread into Europe in the Late Glacial^49^. Some J1c sub-lineages have been also proposed as Early Neolithic founder lineages^5,50^. As for super-haplogroup U, four sub-haplogroups were detected, including U4, the same lineage that pushes modern eastern Umbrians close to central-eastern Europeans in the PCA.

**Figure 5:**
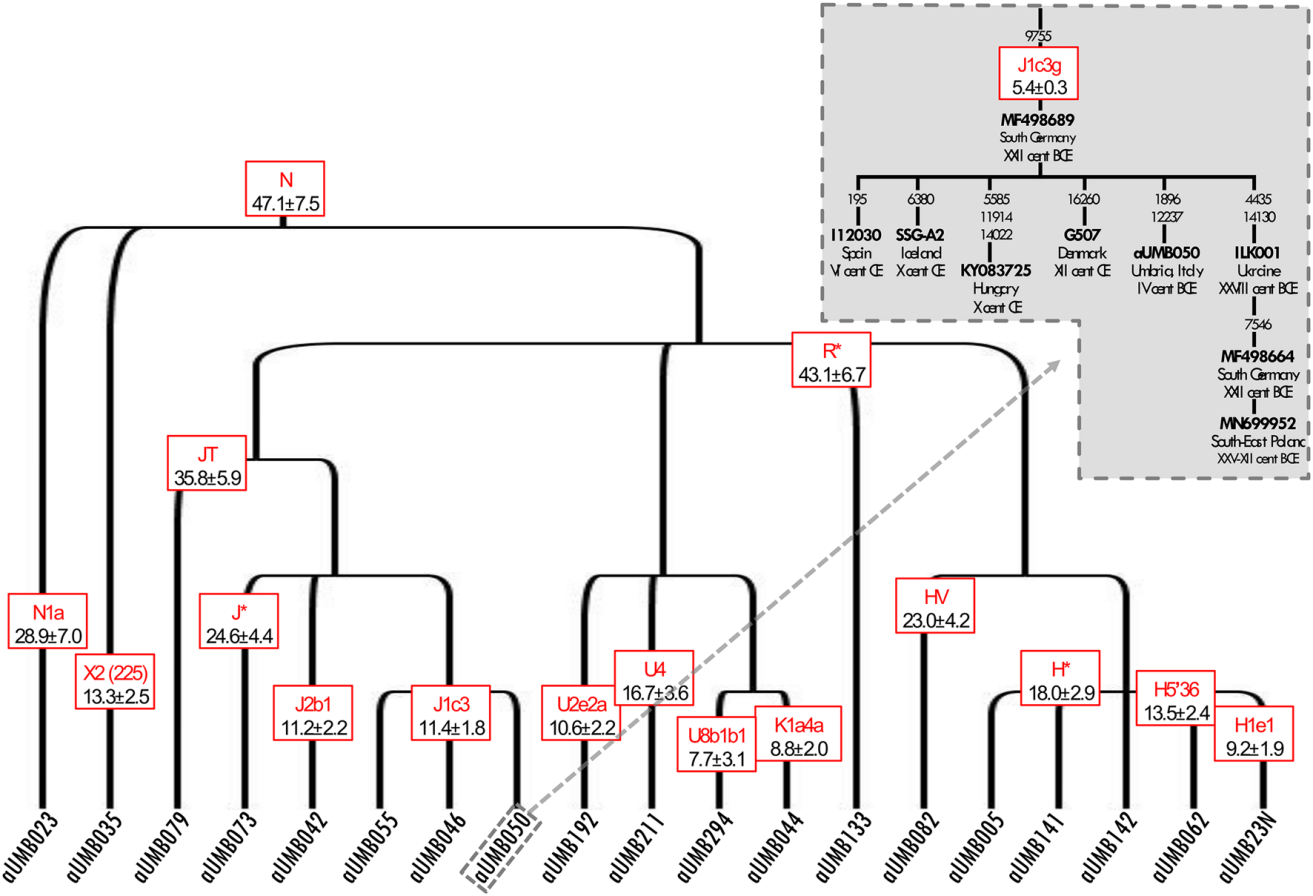
Schematic phylogeny of ancient Umbrians. Bayesian ages refer to the MRCA shared with modern Umbrians. The inset highlights the closeness of the Umbrian J1c3g mtDNA to other available ancient samples.

**Figure 6:**
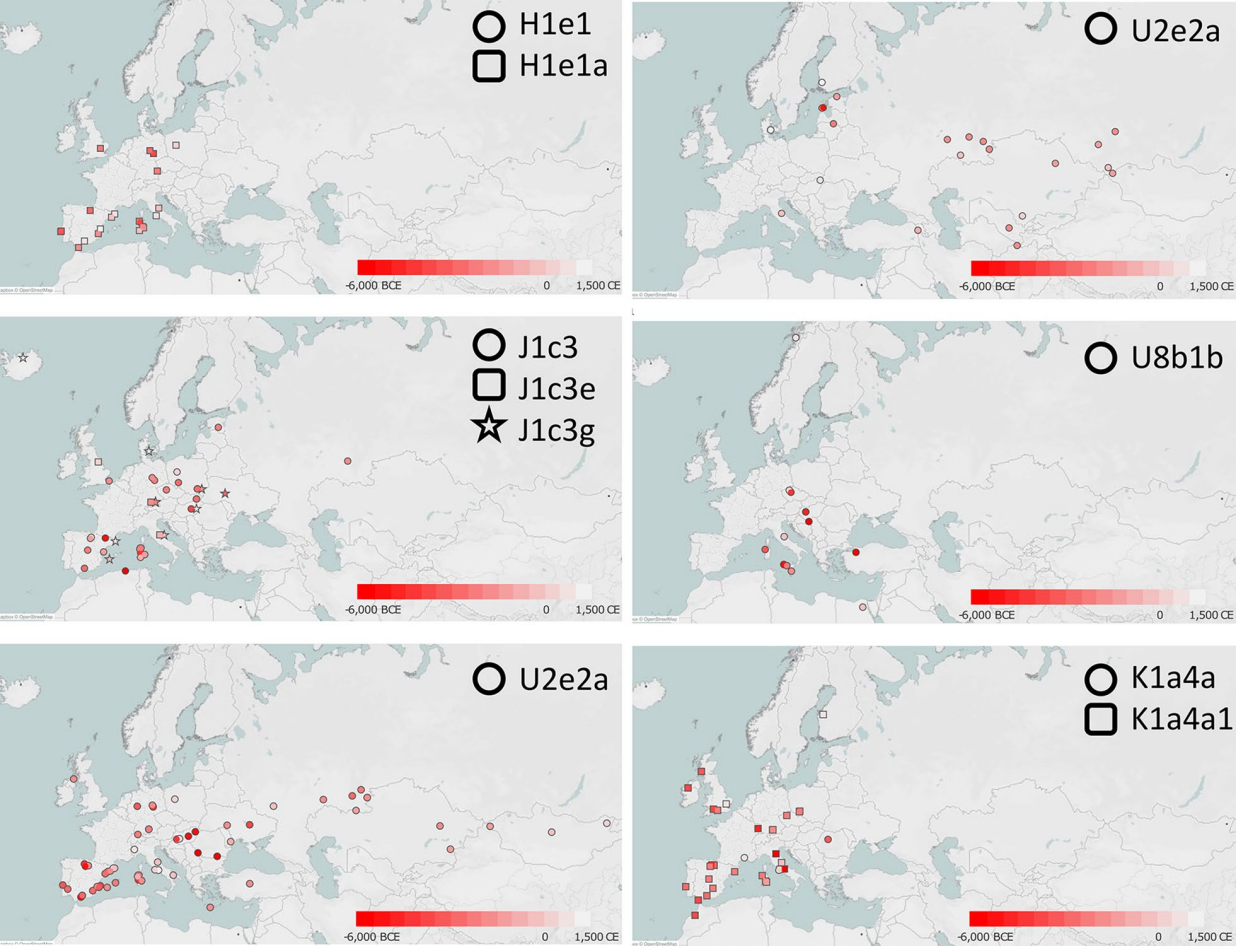
Maps of ancient mitogenomes from literature belonging to the six terminal branches shared between ancient and modern Umbrians. Within each of the six branches, only sub-clades identified in Ancient Umbrians are reported. See Supplementary Dataset S4 for details. Maps were constructed with Tableau 2019.3.0 (www.tableau.com).

The incidence of each major haplogroup identified in our ancient sample is comparable with the one observed in present-day Umbrians (*p* value 0.33). However, the high frequency of haplogroup J in ancient Umbrians (32%) can currently be observed only in the eastern part of the region (30%). Virtually all lineages (except for the paragroups J* and R*) identified in pre-Roman remains are still recognizable nowadays in Umbria, thus suggesting a possible genetic continuity since pre-Roman times (Supplementary Dataset S3). We attempted to verify this continuity on a phylogenetic tree encompassing modern (198) and ancient (19) mitogenomes from Umbria (Fig. 4 and Supplementary Fig. S6). Firstly, the demographic change in the population size depicted by the Bayesian Skyline Plot (BSP) confirms the typical trend of European populations with two sharp increases dated to Paleolithic (from ~ 40 kya) and Neolithic (from ~ 10 kya) ages. Moreover, the age estimates of the major branches overlap with previously reported confidence intervals^50,51^. Even if we did not pinpoint any haplotype identities between modern and ancient samples, about half of the ancient samples share terminal branches (six clades in total: H1e1, J1c3, J2b1, U2e2a, U8b1b1 and K1a4a) with modern Umbrians, all dated back to the Holocene (Figs. 4, 5). We searched public databases for ancient mtDNAs belonging to these lineages identifying 225 ancient mitogenomes from samples excavated in different western Eurasian regions and in northern Africa and dated to prehistoric and historic periods, as shown by the geographic/temporal maps of these sub-lineages (Fig. 6 and Supplementary Dataset S4). J1c3g could be considered a paradigmatic example of these heterogeneous genetic connections, as attested by its aDNA tree, which includes our sample (aUMB050) and other eight ancient mitogenomes from public databases (inset of Fig. 5). Two of these are Bronze Age samples, one from Ukraine^6^ and one from southeastern Poland^52^. Other two burials were excavated in southern Bavaria (Germany), one associated to the early Bronze Age and the other to a Bell Beaker Complex^53^. The latter sample is at the root of the reconstructed J1c3g tree, which has been dated to 5.4 ± 0.3 kya. Four more recent J1c3g mtDNAs have been also identified in one individual from Spain dated to the sixth century CE and archaeologically interpreted as a Visigoth^54^, one Hungarian conqueror^55^ and a pre-Christian Icelander^56^, both from the early tenth century CE, and a medieval sample from Denmark^57^.

## Conclusions

Surrounded by the Mediterranean Sea and bounded by the Alps, Italy extends over more than 1,000 km along a North–South axis and includes the two largest islands of the Mediterranean Sea, Sicily and Sardinia. The combination of this geographic complexity with a rich set of historical events and cultural dynamics had the potential to shape in a unique way the distribution of genetic variation within the Italian populations. Local peculiarities have been highlighted by analyzing the mitogenome variation of specific regions, e.g. Marche, Piedmont, Tuscany and Sardinia^21,36,37,58^. However, a fine and exhaustive microgeographic characterization of other regions has yet to be conducted.

In this study, we describe for the first time the mtDNA variation of the current Umbrian population by analyzing 545 samples covering the entire region. Upon evaluating the genealogical information collected during the sampling campaigns, we reallocated the samples, based on their terminal maternal ancestors, into six sub-areas (north, south, west, center, center-east and east) drawn by geographic criteria and historical/cultural information. A wide range of haplotypes, mostly belonging to western Eurasian haplogroups (97%), testify for the high mtDNA diversity in Umbria. The incidence of these lineages across the region is quite homogeneous with the notable exception of haplogroup K, reaching the highest frequency (17%) in South Umbria, and haplogroup J, which encompasses 30% of current inhabitants of the eastern area. In the western Eurasian PCA plot, the latter sub-region is pushed close to populations from central-eastern Europe by haplogroups U4 and U5a that show high frequencies in those areas.

Then, we extended our analyses to complete mitogenomes (191 sequenced for the first time), randomly selecting the targeted samples to avoid phylogenetic biases and to maintain the population-wide characteristics of our dataset. This higher level of resolution allowed us to refine the haplogroup affiliation in more than 70% of the samples and to make a diachronic comparison with 19 ancient mitogenomes from *Umbri Plestini*. These pre-Roman samples were classified into the same haplogroups identified in contemporary inhabitants. Moreover, the six terminal branches (H1e1, J1c3, J2b1, U2e2a, U8b1b1 and K1a4a) shared between ancient and modern mitogenomes suggest a genetic continuity in the region during the Holocene. These specific lineages were also identified in a wide range of available ancient samples outside the region, including Neolithic Mediterranean remains as well as *Yamnaya*, Bell Beaker and more recent samples from central-eastern Europe. These variegated connections are summarized by the lineage geographic/temporal patterns and are specifically shown by the J1c3g ancient mtDNA tree dated between the Late Neolithic and the Early Bronze Age.

In brief, it is apparent that distinctive mtDNA variants have been brought into the region by the ancestors of *Umbri Plestini* and preserved in some, perhaps more isolated, sub-areas. These ancestors reached Umbria coming from various population sources at different times during the Holocene, from early Neolithic farmers spreading across the Mediterranean to Bronze Age and Medieval connections with central-eastern Europeans, possibly including few nomadic groups (*Yamnaya*) from the Pontic-Caspian steppes. This microgeographic and diachronic mtDNA portrait of Umbria fits well with recent genetic data on the entire peninsula. The Y-chromosome counterpart pointed to different male ancestries for the Italian populations^24^ and the autosomal data revealed several ancient signatures and the largest degree of population structure detected so far in Europe^19,29^. Notably, two of the three published genomic clusters (Sardinia, Northern and Southern Italy) overlap in Central Italy and precisely in Umbria, the “Heart of Italy”. In a wider multidisciplinary context, this hypothesis is also supported by historical sources that list the *Umbri* among the most ancient Italic populations^38–40^ and by the assumed Indo-European origin of their language, distinct from the Etruscan one spoken by neighboring people during the Iron Age^59^.

## Materials and methods

### Modern Umbrians

#### Sample collection

The modern collection consisted of 538 DNA samples from healthy and unrelated subjects with an Umbrian maternal grandmother as a terminal maternal ancestor. Swab or mouthwash rinsing samples were collected from volunteers, representing the entire Umbrian area. Written informed consents were obtained from all donors, who provided information about place of birth and geographical origins up to three generations of Umbrian maternal ancestry. Total DNA was extracted with the MagCore Automated Nucleic Acid Extractor following manufacturer’s protocols. Seven additional Umbrian samples, collected and sequenced in our labs for previous projects^60,61^, were also included.

All analyses were carried out in accordance with relevant guidelines and regulations, and all experimental protocols were approved by the Ethics Committee for Clinical Experimentation of the University of Perugia (protocol no. 2017-01).

#### Geographical division

Umbria was divided into six sub-areas (highlighted in different colors in Fig. 1) considering geographic criteria as well as historical and cultural information. The northern and southern areas are geographically and traditionally linked to Tuscany and Latium, respectively. The hilly lands to the west, including “Monte Peglia” and Orvieto, were part of Etruria. Eastern Umbria is characterized by high mountains (the Apennines) where ancient Umbrians settled for centuries having extensive exchanges with the neighboring Marche populations. Lastly, we decided to divide the vast and flat central area into two sub-regions, here called center and center-east, which are delimited by the Tiber and Topino rivers, respectively. The central area includes cities of known Etruscan origins, such as Bettona, Perugia and Todi. In particular, the name Todi means "border" and, even if it was founded by ancient Umbrians, the city was located at the border with the Etruscan territories and was still under their influence when it was conquered by the Romans. On the contrary, central-eastern Umbria, also known as “Valle Umbra”, includes ancient villages such as Assisi, Bevagna, Spello and the modern municipality of Foligno. Historically, these cities experienced intensive exchanges with eastern Umbria, as testified for instance by two ancient roads, Via Plestina (from Foligno) and Via della Spina (from Spoleto).

#### Control-region sequencing

Novel mitochondrial control-region sequences were generated through standard PCR and Sanger sequencing method^30^, then assembled and aligned to the revised Cambridge Reference Sequence (rCRS; NC_012920.1)^62^ using Sequencher 5.10 (Gene Codes Corporation). These were analyzed together with the control-region sequences from the 191 complete genomes (see below) and seven previously published, for an overall number of 545 control regions (Supplementary Dataset S1).

#### Complete mitogenome sequencing

The entire mitogenome of six present-day samples was sequenced using the classic PCR-Sanger system^63^, while 185 mitogenomes were obtained by employing two Next Generation Sequencing (NGS) techniques: 82 by the Illumina MiSeq^64^ and 103 through the Ion PGM System^65^ (Supplementary Dataset S2).

FASTQ files were aligned to the reference sequence (rCRS; NC_012920.1) using BWA^66^, the bam files were than filtered and sorted with SAMtools^67^. The variants were called employing HaplotypeCaller implemented in GATK (with ploidy flag set as 1)^68^ and filtered using BCFtools to obtain the final SNP dataset. Three different in-house scripts (HeteroSeek, HaploCreate and HaploCreateBellow, developed at the IPATIMUP Institute) were used to obtain the final haplotypes (both with and without heteroplasmies). The final haplotypes were also double-checked through a manual visualization of the bam files with the Integrative Genomics Viewer (IGV) software. Common criteria used for calling mtDNA variants were adopted as reported by Olivieri and colleagues^58^. In addition, some problematic fragments were replicated by Sanger sequencing and the congruence with the initial control-region data was evaluated.

### Ancient Umbrians

#### Ancient sample collection

We analyzed the remains of 28 individuals excavated from the necropolis of *Plestia* in Colfiorito (East Umbria, Central Italy, Fig. 1), in which more than 250 tombs have been identified. According to funerary rites and grave goods, the necropolis was dated from the early nineth to the late third century BCE and provided a greater understanding of the life and culture of the ancient Umbrian civilization (see Supplementary Figure S1 and Supplementary Text for further details). Direct radiocarbon dating on the skeletal remains of four individuals was performed in outsourcing at the Curt-Engelhorn-Centre for Archaeometry (Mannheim, Germany).

#### Ancient mitogenome sequencing

Molecular analysis of the archaeological specimens was performed under sterile conditions in a dedicated ancient DNA (aDNA) facility at the Laboratory of Molecular Anthropology and Paleogenetics (University of Florence, Italy), following strict guidelines and standard precautions to avoid contaminations. After a silica-based DNA extraction^69^ and libraries preparation^70^, ancient mitogenomes were captured and sequenced on the Illumina MiSeq platform at the Institute of Biomedical Technologies, National Research Council (Segrate, Milano, Italy), as previously reported^71^.

After demultiplexing, raw reads were analyzed using a specific pipeline developed for aDNA. The EAGER pipeline^72^ was used for initial sequencing quality control, adapter trimming and paired-end read merging. Merged reads were filtered for a minimum length of 30 base pairs and mapped to rCRS (NC_012920.1) using CirculaMapper (BWA parameters: − n 0.02, − l 16,500), a tool integrated in EAGER and specifically designed for the analysis of circular reference genomes. After removing PCR duplicate, only reads with a map quality score ≥ 30 were retained and used for reconstructing mtDNA consensus sequences using *schmutzi* (parameters: − logindel 1 − uselength)^46^. Bases with individual likelihood < 20 were considered as unassigned positions (Ns). Present-day human contamination was evaluated by an iterative likelihood method implemented in *schmutzi* using a non-redundant database of 197 human mitochondrial genomes available in the software package. Damage patterns at the ends of the molecules were calculated using contDeam, a program provided with the *schmutzi* package.

#### Phylogenetic and statistical methods

Several mtDNA sequence variation parameters were estimated using DnaSP 5.1 software^73^. Intra- and inter-population comparisons based on the number of pairwise differences between sequences were performed using an Arlequin integrated R script^74^.

Haplogroups were predicted using HaploGrep2 software^75^, but the initial classification was revised and manually updated in agreement with PhyloTree build 17^76^ and SAM 2^77^ on EMPOP^78^.

All (modern and ancient) haplotypes underwent a posteriori mtDNA sequence data quality control using EMPcheck, a tool to perform plausibility checks on a rCRS-coded data table (https://empop.online/tools).

In order to graphically display (and summarize) the relationships among the analyzed mtDNAs, Principal Component Analyses (PCA) were also performed using Excel software implemented by XLSTAT, as previously described^30^. Spatial frequency distribution plots were constructed with the program Tableau 2019.3.0. Finally, after purging all positions containing gaps and ambiguous data, a maximum parsimony tree was built with mtPhyl v.5.003, while time estimates and demographic trends were evaluated using BEAST v2.6.1 (Bayesian Evolutionary Analysis of Sampling Trees), as previously reported^58^.

## Supplementary information

Supplementary file1

Supplementary file2

Supplementary file3

## Data Availability

All novel sequences have been deposited in GenBank under accession numbers: MN686759-MN687105 for 347 mitochondrial control-region sequences from modern samples; MN687107-MN687297 for 191 complete mitochondrial sequences from modern samples; MN687298-MN687316 for 19 complete mitochondrial sequences from ancient samples. The data will be available from the EMPOP mtDNA population database (https://empop.online/) under accession numbers EMP00826 (control-region data) and EMP00827 (mitogenomes).
