# Supplementary material for: The mitogenome portrait of Umbria in Central Italy as depicted by contemporary inhabitants and pre-Roman remains
Source: Sci Rep. 2020 Jul 1;10:10700. doi: 10.1038/s41598-020-67445-0 (PMC7329865; doi:10.1038/s41598-020-67445-0)
Supplement: Supplementary file 1 — Supplementary file1 [file 41598_2020_67445_MOESM1_ESM.docx]

**Supplementary Text**

**The mitogenome portrait of Umbria in Central Italy as depicted by contemporary inhabitants and pre-Roman remains**

**Alessandra Modi^1#^, Hovirag Lancioni^2#^*, Irene Cardinali^2#^, Marco R. Capodiferro^3#^, Nicola Rambaldi Migliore^3^, Abir Hussein^3^, Christina Strobl^4^, Martin Bodner^4^, Lisa Schnaller^4^, Catarina Xavier^4^, Ermanno Rizzi^5^, Laura Bonomi Ponzi^6^, Stefania Vai^1^, Alessandro Raveane^3^, Bruno Cavadas^7,8^, Ornella Semino^3^, Antonio Torroni^3^, Anna Olivieri^3^, Martina Lari^1^, Luisa Pereira^7,8^, Walther Parson^4,9^, David Caramelli^1^, Alessandro Achilli^3^***

^1^Department of Biology, University of Florence, Florence, 50122, Italy;

^2^Department of Chemistry, Biology and Biotechnology, University of Perugia, Perugia, 06123, Italy;

^3^Department of Biology and Biotechnology “L. Spallanzani”, University of Pavia, Pavia, 27100, Italy;

^4^Institute of Legal Medicine, Medical University of Innsbruck, Innsbruck, 6020, Austria;

^5^Istituto di Tecnologie Biomediche, CNR, Segrate, Milano, 20090, Italy;

^6^M.A.N.U. National Archeological Museum of Umbria, Perugia, 06121, Italy;

^7^IPATIMUP (Instituto de Patologia e Imunologia Molecular da Universidade do Porto), Porto, Portugal;

^8^i3S (Instituto de Investigação e Inovação em Saúde, Universidade do Porto), Porto, 4200-135, Portugal;

^9^Forensic Science Program, The Pennsylvania State University, University Park, PA, 16801, USA.

^#^ Equal contribution

*** Correspondence:**Hovirag Lancioni

hovirag.lancioni@unipg.it

Alessandro Achilli

alessandro.achilli@unipv.it

**Conflict of interest**

The authors declare that the research was conducted in the absence of any commercial or financial relationships that could be construed as a potential conflict of interest.

**The necropolis of *Plestia* (East Umbria)**

The necropolis of *Plestia* extends on a plateau that represented the so-called via *Plestina*. The excavation campaigns, with more than 250 inhumations graves excavated until now, involved a large area of about 12 hectares. The tombs are arranged in groups, following a circular path, like other Umbrian necropolis and peculiar to the Umbrian funerary traditions^1^. The bodies are often supine and placed directly on the ground. Burials are mostly single, but some of them were double and triple, thus not considered for the genetic analyses.

The necropolis covers a period ranging from the beginning of the 9^th^ century to the 3^rd^ century BCE. Archaeological and topographic analyses allowed referring the tombs to four chronological phases, all to be traced back to the Italic population of the proto- and pre-Roman *Plestini*.

- Phase I (9^th^ - 7^th^ centuries BCE) suggests a non-stratified society that buried its members with grave goods of local manufactures, as in graves 39 (dated back to the most ancient period), 5 and 211, the latter dating more precisely to the second half of the VIII century BCE. Ceramics and other ornamental objects show close connections with the area of Terni, Latium and Southern Etruria (Cerveteri, Tarquinia and especially Veio), southern Italy (Cuma, Sala Consilina), and with the Sabina area (Sabine Hills around the Nera river). Those goods include long-distance products, like metals and amber, but also more restricted salt-trade for the internal areas of Central Italy.
- Phase II (7^th^ and 6^th^ centuries BCE) to which the feminine grave 141 probably belongs.
- Phase III (beginning of the 6^th^ up to the 4^th^ centuries BCE): represented by tombs 5 and 133, relative to the initial period, and 8, 23, 42, 46, 50, 62, 73, 142 and 192, relative to the second period. Despite the timespan, these graves testify for a homogeneous context, characterized by a demographic growth, an enrichment of outfits (both ceramic and metal vases) and a different burial arrangement (with goods lateral to the body). The social differences are now evident. Among imported goods, we can notice the first appearance of Attic ceramics, which coincides with the emergence of Numana as a thriving commercial center and a probable sorting point for the trade of *Plestia*. Both qualitatively and quantitatively relevant are the imports from Etruria. Overall, it emerges a sudden Etruscan expansion (*Volsinii*, in particular) towards the Po Valley and Campania. This is the phase in which the culture of *Plestia* is unequivocally highlighted, within a large area delimitated by the Topino valley, Mount Subasio, Menotre and Nera valleys and Mount Pennino.
- Phase IV (late 4^th^ and 3^rd^ centuries BCE), to which the grave 109 belongs, represents the terminal phase of the necropolis, characterized by a limited number of tombs (24), and by the simplification and depletion of funeral goods both in qualitative and quantitative terms. It witnessed a demographic tightening, and a decreasing of relations with the Etruscan world, replaced by Faliscan productions. The historical events that involved Central Italy in this period, such as the Gallic wars, the Roman expansion and the second Punic war, which in this area records the episode of the battle near the Lake *Plestia*, contributed to the decline of *Plestia* civilization, which lost its culture and identity and was assimilated to the Roman Empire.

In summary, it emerges a dynamic picture of central Italy, which was quite homogenous from different points of views (cultural, political, social and economic) and led to the development of distinct autonomous entities, such as *Plestia*^1^. Even if the archaeologists could not establish the origin of the group that at the beginning of the 9^th^ century BCE chose to settle on the Colfiorito plateau, probably there was a displacement of one population from nearby areas, such as Nocera Umbra or Pieve Torina with attested late Bronze Age settlements, then abandoned. This phenomenon was also observed in other surrounding areas (e.g. from Piediluco to Terni). The other hypothesis points to the southern area called Sabina, considering the close affinities with Terni, Tivoli, etc.. These hypotheses are still valid and not mutually exclusive.

**Reference**

1 Bonomi Ponzi, L. *La Necropoli Plestina di Colfiorito di Foligno*. (Quattroemme, 1997).
