# Supplementary material for: The mitogenome portrait of Umbria in Central Italy as depicted by contemporary inhabitants and pre-Roman remains
Source: Sci Rep. 2020 Jul 1;10:10700. doi: 10.1038/s41598-020-67445-0 (PMC7329865; doi:10.1038/s41598-020-67445-0)
Supplement: Supplementary file 3 — Supplementary file3 [file 41598_2020_67445_MOESM3_ESM.docx]

**Supplementary figures S1-S5**

**The mitogenome portrait of Umbria in Central Italy as depicted by contemporary inhabitants and pre-Roman remains**

**Alessandra Modi^1#^, Hovirag Lancioni^2#^*, Irene Cardinali^2#^, Marco R. Capodiferro^3#^, Nicola Rambaldi Migliore^3^, Abir Hussein^3^, Christina Strobl^4^, Martin Bodner^4^, Lisa Schnaller^4^, Catarina Xavier^4^, Ermanno Rizzi^5^, Laura Bonomi Ponzi^6^, Stefania Vai^1^, Alessandro Raveane^3^, Bruno Cavadas^7,8^, Ornella Semino^3^, Antonio Torroni^3^, Anna Olivieri^3^, Martina Lari^1^, Luisa Pereira^7,8^, Walther Parson^4,9^, David Caramelli^1^, Alessandro Achilli^3^***

^1^Department of Biology, University of Florence, Florence, 50122, Italy;

^2^Department of Chemistry, Biology and Biotechnology, University of Perugia, Perugia, 06123, Italy;

^3^Department of Biology and Biotechnology “L. Spallanzani”, University of Pavia, Pavia, 27100, Italy;

^4^Institute of Legal Medicine, Medical University of Innsbruck, Innsbruck, 6020, Austria;

^5^Istituto di Tecnologie Biomediche, CNR, Segrate, Milano, 20090, Italy;

^6^M.A.N.U. National Archeological Museum of Umbria, Perugia, 06121, Italy;

^7^IPATIMUP (Instituto de Patologia e Imunologia Molecular da Universidade do Porto), Porto, Portugal;

^8^i3S (Instituto de Investigação e Inovação em Saúde, Universidade do Porto), Porto, 4200-135, Portugal;

^9^Forensic Science Program, The Pennsylvania State University, University Park, PA, 16801, USA.

^#^ Equal contribution

*** Correspondence:**Hovirag Lancioni

hovirag.lancioni@unipg.it

Alessandro Achilli

alessandro.achilli@unipv.it

**Conflict of interest**

The authors declare that the research was conducted in the absence of any commercial or financial relationships that could be construed as a potential conflict of interest.

**Supplementary Figure S1**

**Supplementary Figure S1. Archaeological information**. Map of the Colfiorito archaeological site showing the distribution of the graves within the necropolis (modified from^1^) and archaeological information of the 28 ancient Umbrians sampled for DNA analysis. The colors of the graves symbolize different chronological phases. In the left box, an example of a burial scheme with skeletal remains and funerary goods distribution.

**Supplementary Figure S2**

**Supplementary Figure S2. Haplotype diversity comparison.** Haplotype diversity comparison between our Umbrian samples and a dataset of 79 modern and ancient populations from Europe, North Africa, the Near East and Central Asia (comparative values from^2^). Dots represent the haplotype diversity distributed along the x-axis proportional to the number of individuals of each population.

**Supplementary Figure S3**


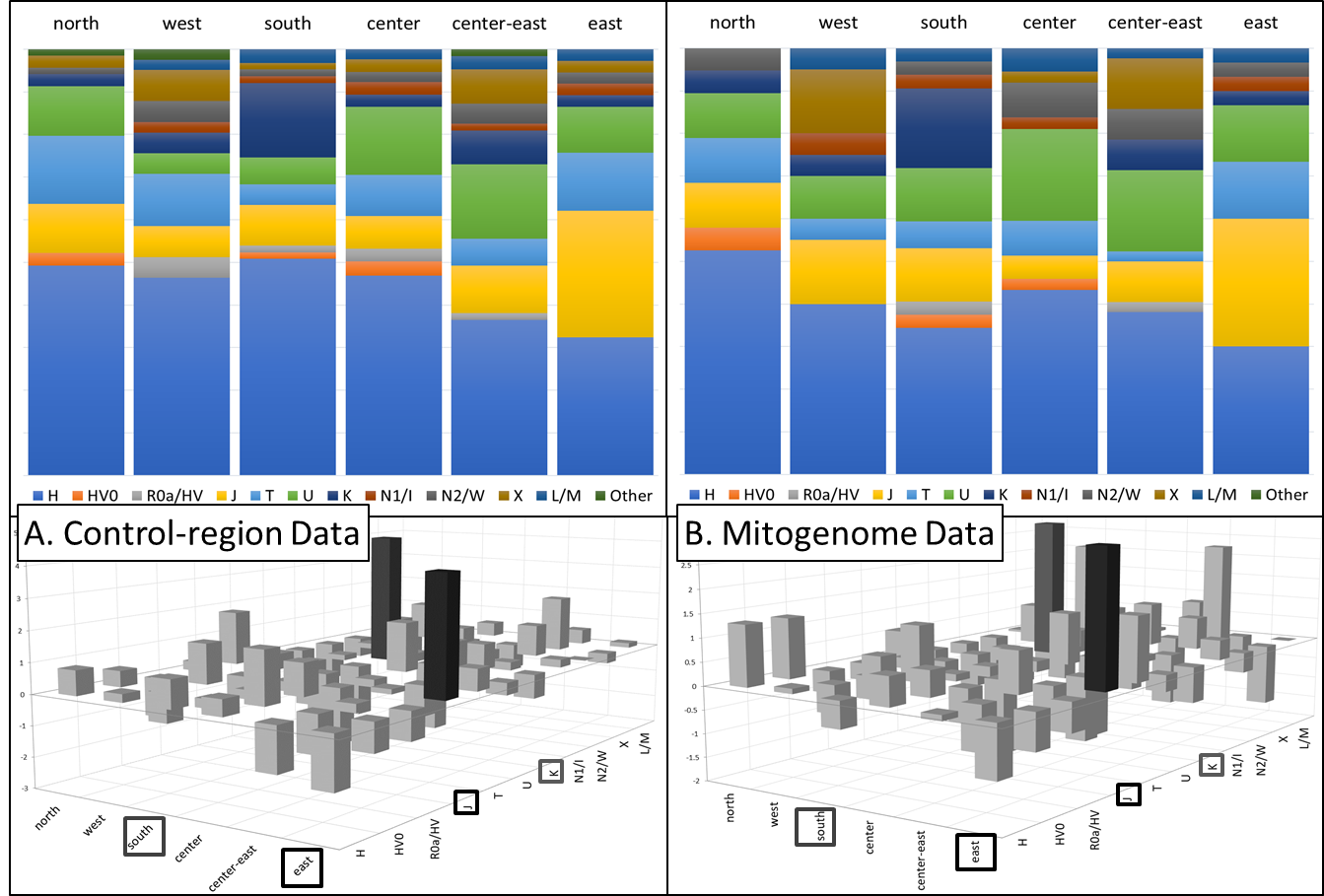


**Supplementary Figure S3. Frequency of haplogroups in modern Umbrians of different geographic sub-regions.** Stacked bar charts show the super-haplogroup (from Haplogrep) relative frequencies in the six Umbria sub-regions based on control-region (A) and complete mitogenome (B) data. The 3D bar charts (below) indicate the contribution of each super-haplogroup to the chi-square test per cell (adjusted residual). Black bars indicate statistical significance at α=0.05.

**Supplementary Figure S4**

**Supplementary Figure S4. SFS plot.** Site Frequency Spectrum (SFS) analysis on the modern Umbrian complete mitogenomes considering the different NGS methods. No significant differences were observed.

**Supplementary Figure S5**

**
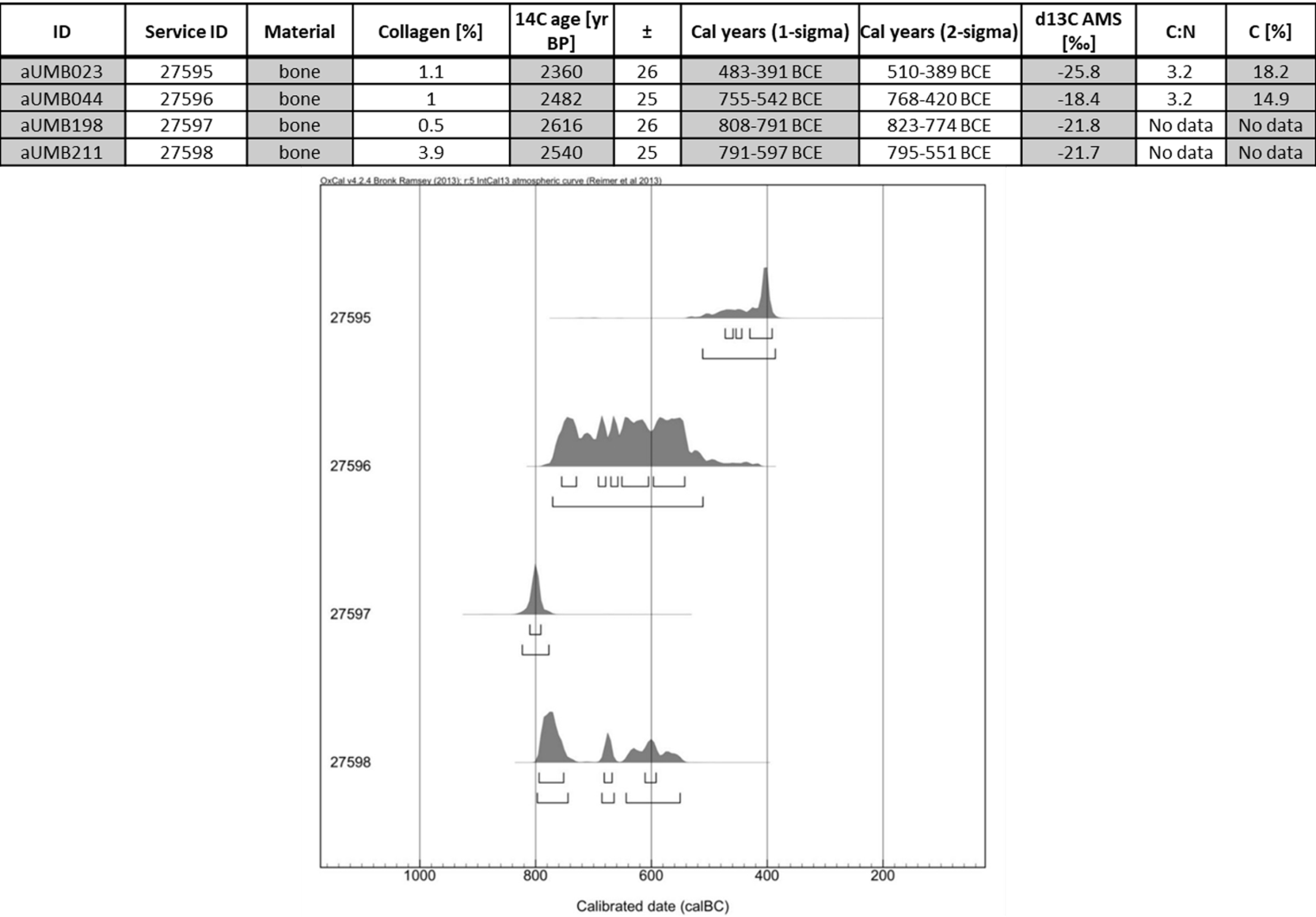
**

**Supplementary Figure S5. Radiocarbon dating of four ancient Umbrians.** The ^14^C ages are given in years BP (years before 1950). These values were converted into calendar ages using the dataset INTCAL13^3^ and the software SwissCal 1.0 (L. Wacker, ETH-Zürich, Switzerland). The results of the calibration are shown in columns “Cal 1-sigma” and “Cal 2-sigma” using the 1-sigma and 2-sigma uncertainty of the ^14^C ages, respectively. Calibration plots were created with OxCal 4.2.

**References**

1 Bonomi Ponzi, L. *La Necropoli Plestina di Colfiorito di Foligno*. (Quattroemme, 1997).

2 Vai, S. *et al.* Genealogical relationships between early medieval and modern inhabitants of Piedmont. *PLoS One* **10**, e0116801, doi:10.1371/journal.pone.0116801 (2015).

3 Reimer, P., Bard, E., Bayliss, A., Beck, J, Blackwell, P., Ramsey, B., et al. IntCal13 and Marine13 Radiocarbon Age Calibration Curves 0–50,000 Years cal BP. *Radiocarbon* **55**, doi:10.2458/azu_js_rc.55.16947 (2013).
